# Prediction of Disease-related microRNAs through Integrating Attributes of microRNA Nodes and Multiple Kinds of Connecting Edges

**DOI:** 10.3390/molecules24173099

**Published:** 2019-08-26

**Authors:** Ping Xuan, Lingling Li, Tiangang Zhang, Yan Zhang, Yingying Song

**Affiliations:** 1 School of Computer Science and Technology, Heilongjiang University, Harbin 150080, China; 2 School of Mathematical Science, Heilongjiang University, Harbin 150080, China

**Keywords:** miRNA–disease associations, projection of node attributes, nonnegative matrix factorization, projection of connecting edges, low-dimensional feature vector

## Abstract

Identifying disease-associated microRNAs (disease miRNAs) contributes to the understanding of disease pathogenesis. Most previous computational biology studies focused on multiple kinds of connecting edges of miRNAs and diseases, including miRNA–miRNA similarities, disease–disease similarities, and miRNA–disease associations. Few methods exploited the node attribute information related to miRNA family and cluster. The previous methods do not completely consider the sparsity of node attributes. Additionally, it is challenging to deeply integrate the node attributes of miRNAs and the similarities and associations related to miRNAs and diseases. In the present study, we propose a novel method, known as MDAPred, based on nonnegative matrix factorization to predict candidate disease miRNAs. MDAPred integrates the node attributes of miRNAs and the related similarities and associations of miRNAs and diseases. Since a miRNA is typically subordinate to a family or a cluster, the node attributes of miRNAs are sparse. Similarly, the data for miRNA and disease similarities are sparse. Projecting the miRNA and disease similarities and miRNA node attributes into a common low-dimensional space contributes to estimating miRNA-disease associations. Simultaneously, the possibility that a miRNA is associated with a disease depends on the miRNA’s neighbour information. Therefore, MDAPred deeply integrates projections of multiple kinds of connecting edges, projections of miRNAs node attributes, and neighbour information of miRNAs. The cross-validation results showed that MDAPred achieved superior performance compared to other state-of-the-art methods for predicting disease-miRNA associations. MDAPred can also retrieve more actual miRNA-disease associations at the top of prediction results, which is very important for biologists. Additionally, case studies of breast, lung, and pancreatic cancers further confirmed the ability of MDAPred to discover potential miRNA–disease associations.

## 1. Introduction

MicroRNAs (miRNAs) are small noncoding, single-stranded RNAs encoded by endogenous genes with a length of approximately 22–24 nucleotides [1,2,3,4]. MiRNAs play important regulatory roles by targeting messenger RNA for splicing or translational inhibition in animals and plants [5]. Increasing evidences shows that miRNAs are involved in the development and progression of many diseases [6,7,8,9]. Therefore, identifying the regulatory relationships between diseases and miRNAs can help researchers explore the pathogenesis of disease.

Early studies mainly used biological experiments to obtain high-accuracy experimental results that fundamentally proved the associations of miRNAs and diseases. However, experimental methods are costly and time-consuming and have low success rates. In recent years, researchers have increasingly turned to computational biology to predict disease miRNAs, which has achieved good results. Our previous work can be divided into two categories. The first category [10,11,12,13] is the inference of candidate diseases based on the regulatory relationships of miRNAs and target miRNAs. Since the number of experimentally validated target miRNAs is insufficient, a set of putative targets is typically inferred by a prediction program. Next, we use the target miRNA and genes associated with known diseases to calculate miRNAs similarities. However, the results of the prediction program have high false-positive rates, reducing the performance of such methods.

The second kind of method is mainly based on miRNAs with similar functions that are typically associated with similar diseases, which is useful for predicting disease-related candidates [14,15,16,17,18]. First, Wang et al. [19] used miRNA-associated diseases to calculate miRNAs similarities. Previous studies were conducted to build miRNA networks based on miRNAs similarities and random walking around the network to obtain network topology information [20,21,22] to infer miRNA–disease associations. Some methods used miRNA similarities to model nonnegative matrix factorization [23,24,25] to predict diseases miRNAs. These methods rely on specific diseases associated with related known miRNAs and do not apply to new diseases without related known miRNAs. In a heterogeneous network with information of disease similarities, miRNA similarities, and miRNA–disease associations, there are many different methods for predicting disease-related candidates. Some methods use machine learning methods [26,27,28] such as ensemble learning [29] to predict disease-associated miRNAs. For example, path information [30] has been used in heterogeneous networks to predict associations between diseases miRNAs and candidate miRNAs associated with diseases could be predicted by matrix factorization or random walks on heterogeneous networks [31]. However, most methods do not consider the node attributions of miRNA or low-dimensional projection representation of miRNAs and diseases.

Rfam [32] incorporated multiple miRNA with similar mature miRNA sequences into the same miRNA family through multi-sequence alignment. There is a consistent seed region among miRNAs in the same family. The seed region refers to the 2–8 bases at the 5′ end of a mature miRNA, which is the key region for the interaction between a miRNA and target gene. Therefore, miRNAs belonging to the same family may regulate similar target genes and thus may be associated with similar diseases. Previous studies showed that some human miRNAs are distributed very close to each other in the genome (<20 kb), i.e., they are distributed in clusters. Multiple miRNAs belonging to the same cluster typically transcribe synchronously and perform certain functions in coordination. Thus, miRNAs in the same cluster are more likely to be associated with similar diseases. Therefore, obtaining information on the encoding of families and clusters of miRNAs is necessary [33,34]. Based on miRNA node attributions, we can project miRNA similarities matrix, disease similarities matrix, and miRNA node attributions to obtain a representative low-dimensional space. Previous approaches to integrating miRNA families and cluster information did not project such information into low-dimensional feature spaces. The advantage of projection is that it extracts representative information on low-dimensional features, which in turn helps to improve predictive disease-associated miRNA performance.

We propose MDAPred, a new method for predicting the associations of candidate disease miRNAs. MDAPred integrates the node attributes of miRNAs and the related similarities and associations of miRNAs and diseases. MDAPred deeply integrates the projection of information such as miRNAs, diseases, miRNA families, and clusters in low-dimensional feature spaces. Projecting miRNAs and diseases and miRNA node attributions into a common low-dimensional space is useful for measuring the distance between miRNAs and diseases. The distance is closely related to the association of the miRNA with the disease. Because miRNAs with similar neighbours are more likely to be associated with similar diseases, the model makes full use of the miRNA’s neighbour information. Thus, a predictive model based on various projections and miRNA neighbour information was built and an iterative algorithm was developed to solve the model to obtain predictions of the associations of miRNAs and diseases. Experimental results based on cross-validation showed that MDAPred method has superior performance compared to several other state-of-the-art methods. Particularly, when focusing on the top part of the prediction results, MDAPred method successfully retrieved more real disease miRNAs. The case studies of three cancers further confirmed the ability of MDAPred to discover potential miRNA–disease associations.

## 2. Results and Discussion

### 2.1. Evaluation Metrics

We used 5-fold cross-validation as an evaluation method for predicting the miRNA and disease association performances. We randomly divided the associations of all known disease miRNAs into five equal parts. Of these, 4 were training sets for training the models and the remaining one was used as a test set for evaluation. We regarded the association in the test set as a positive sample and association between all unobserved miRNAs and diseases as a negative sample. In our association prediction ranking, a higher ranking of positive samples indicated better prediction performance.

Using a model based on nonnegative matrix factorization, we obtained predicted scores for miRNAs and diseases and ranked them in descending order. In this descending order, a higher positive example indicated better the prediction performance. For a pair of known associated diseases and miRNAs, if the association prediction score obtained by the model is higher than the threshold δ we set, it is judged as a positive sample. Otherwise, if the predicted score of the counter example is lower than δ, the sample is judged as negative. By varying the size of threshold δ, the corresponding true-positive rate (TPR) and false-positive rate (FPR) can be obtained and are defined as follows,

(1) $$TPR=\frac{TP}{TP+FN},\;FPR=\frac{FP}{TN+FP},$$

where *TP* is the number of positive samples, *TN* is the number of the negative samples, and *FN* is the number of positive samples misidentified as negative. Correspondingly, *FP* indicates the number of negative samples misidentified as positive. TPR indicates the proportion of positive samples correctly identified among the total positive samples, and FPR is the misidentified negative samples accounting for all negative samples. By changing the threshold δ, we can obtain different TPR and FPR values. These TPR and FPR were used to plot the receiver operating characteristic (ROC) curve. The overall predicted performance was evaluated by calculating the area under the ROC curve (AUC).

Since the ratio of the number of unobserved miRNA–disease associations (negative samples) to the number of known associations (positive samples) was 1:30, there was a serious class imbalance between the positive and negative samples. Therefore, we used the precision-recall (PR) curve, which is more convincing than the ROC curve [35], as another evaluation standard. Similarly, by changing the threshold, new precision and recall values can be obtained to draw the PR curve and the area of PR curve (AUPR) is calculated. The precision and recall values are defined as follows,

(2) $$Precision=\frac{TP}{TP+FP},\;Recall=\frac{TP}{TP+FN},$$

where precision refers to the proportion of correctly recognized positive examples in the retrieved samples, while recall represents the ratio of correctly recognized positive examples to the total number of positive examples.

Additionally, biologists typically select the top miRNA candidates in the prediction results to verify their associations with diseases through biological experiments. In the prediction results of the top *k*, a larger number of positive samples appear to indicate more valuable predictions. Therefore, we calculated the recall rate of the top *k*, which is the ratio of positive samples in top *k* relative to the total positive samples, as another criterion for evaluating disease and miRNA performance.

Currently, the data for miRNA and disease association showed that most diseases are only associated with a few miRNAs, leading to a lack of sufficient association data to evaluate prediction models. Therefore, we selected 15 common diseases from the database for cross-validation and simulation experiments, each with a well-characterized disease and typically associated with at least 80 miRNAs.

### 2.2. Comparison with Other Methods

To better evaluate the predictive performance of MDAPred, we compared the method to GSTRW [22], BNPMDA [36], Liu’s method [37], PBMDA [30], and DMPred [23] as state-of-the-art methods for predicting miRNA and disease associations. We adjusted the hyperparameters of these comparison methods to achieve the best prediction performance. Based on the results of a cross-validation analysis, the value of hyperparametric $\alpha_{1},\;\alpha_{2},\;\alpha_{3},\;and\;\alpha_{4}$ of MDAPred was selected from $\;\left\{0.01,\;0.1,\;1,\;10\right\}$. MDAPred showed the best performance when $\alpha_{1}=0.1,$ $\alpha_{1}=0.1,$ $\alpha_{2}=0.1$, $\alpha_{3}=0.1$, and $\alpha_{4}=0.1$. For the comparison method, we based the hyperparameters on the best parameters in the corresponding papers (γ=θ=0.2, α=β=0.8, ω=0.6 for GSTRW; λM=17, λD=110, θ=120 for DMPred; λ=0.8, δ=0.9, η=0.1, λ=0.5 for Liu’s method; L=3, α=2.26 for PBMDA, and α=0.5, β=0.5 for BNPMDA). 

**Figure 1:**
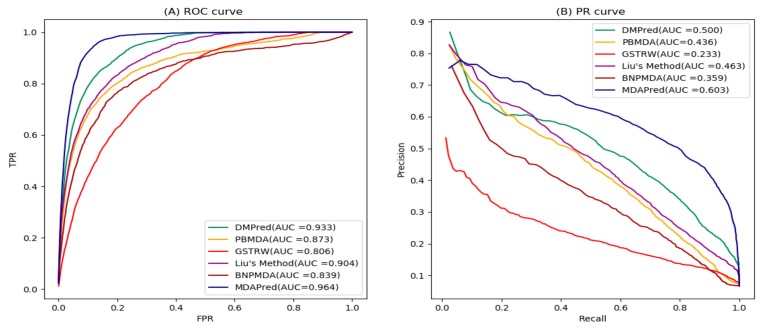
Receiver operating characteristic (ROC) and precision-recall (PR) curves of MDAPred and the other five methods. (**A**) ROC curves (**B**) PR curves.

DMPred exploited nonnegative matrix factorization to predict candidate miRNAs and achieved better performance. You et al. proposed a method called PBMDA which inferred disease-related miRNA by exploiting the information of paths connecting miRNAs and disease. GSTRW is a prediction miRNA–disease association method based on random walk. Liu’s method inferred potential candidate miRNAs by exploiting the network topology information. BNPMDA predicted disease-related miRNA based on hierarchical clustering. Figure 1 demonstrates the receiver operating characteristic (ROC) and precision-recall (PR) curves of MDAPred and the other five methods.

As shown in Figure 1A and Table 1, MDAPred method achieved the best average performance (AUC = 0.964) among all 15 diseases that we considered. In particular, it outperformed DMPred by 3.1%, PBMDA by 9.1%, GSTRW by 15.8%, Liu’s method by 6.0%, and BNPMDA by 12.5%. We also listed the AUC of all six methods on 15 well-characterized human disease (Table 1), MDAPred yielded the best performed for 13 of the common diseases. GSTRW used disease similarities and miRNA similarities when predicting the candidate miRNAs but did not consider the disease miRNA associations. Therefore, GSTRW showed the lowest performance. As shown in Figure 1A, the ROC curves of both BNMPDA and PBMDA overlapped. PBMDA using path information performed better than the BNMPDA using layer clustering. Liu’s method achieved better results than the above two methods. Although these methods use different calculations, they make full use of the topology information of heterogeneous networks. DMPred based on nonnegative matrix factorization used network topology and the original features of miRNAs and diseases for predicting associations, which achieved a competitive prediction performance. MDAPred is also based on a nonnegative matrix algorithm. Unlike DMPred, this method considers not only node attributes but also uses projection to obtain the association prediction. Figure 1A and Table 1 show that MDAPred exhibited the best performance against 15 common diseases.

As shown in Figure 1B, the average PR curve of the 15 common diseases of MDAPred was higher than that of the other five methods. The average AUC of MDAPred was 10.3% better than DMPred, 16.7% better than PBMDA, 38% better than GSTRW, 14% better than Liu’s method, and 24.4% better than BNPMDA. Of the 15 common diseases, MDAPred showed the best performance in 14 of these diseases (Table 2).

**Figure 2:**
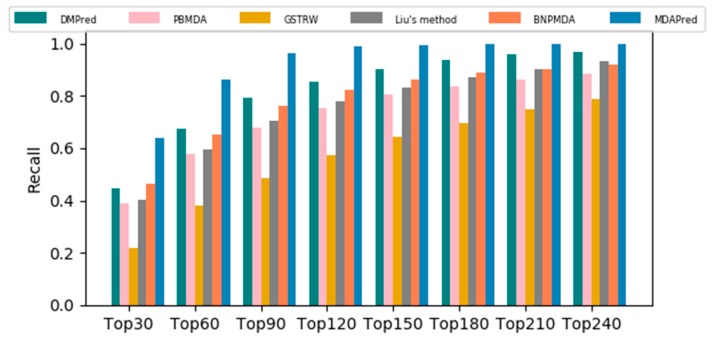
Recall rates of 15 diseases under different top *k*.

A higher recall rate of the top *k* of miRNAs indicates that more true miRNAs associated with diseases are correctly identified. The top *k* average recall rate for 15 common diseases is shown in Figure 2. Under the various top *k*, MDAPred method recall was significantly higher than those of the other methods. For the top 30, MDAPred method showed a recall rate of 0.641, the top 60 recall rate was 0.862, and the top 90 recall rate was 0.965. The recall rate of the top 30 for DMPred method was 0.448, for the top 60 was 0.675, and for the top 90 was 0.791. Most recall values determined using PBMDA were close to those obtained using Liu’s method. The former’s top 30, top 60, and top 120 call values were 0.390, 0.580, and 0.680, respectively. The latter’s top 30, top 60, and top 120 call values were 0.402, 0.594, and 0.705, respectively. BNPMDA’s top 30, top 60, and top 90 were 0.465, 0.653, and 0.764 respectively. GSTRW method showed the worst performance, with a top 240 recall value of only 0.79.

In addition, to further verify that the AUCs and AUPRs of MDAPred were significantly higher than those of other methods, we perform a paired *t*-test. All paired *t*-test results were less than 0.05, which indicates that MDAPred’s performance was significantly better than that of other methods (Table 3).

### 2.3. Case Studies

To demonstrate the ability of MDAPred to discover high-quality candidate miRNAs, we conducted case studies of breast, pancreatic, and lung cancers. Because breast cancer is one of the most common cancers, we used it as an example to analyze its top 50 candidates in detail (Table 4).

Xie et al. used text mining techniques to extract the association between experimentally validated miRNAs and diseases [38]. These associations were further manually verified and have been incorporated into miRCancer database, which contains 632 cancer-associated 6323 miRNA–disease associations. dbDEMC [39] is a differentially expressed miRNA database in human cancers containing 2224 miRNAs differentially expressed in 36 cancers. As shown in Table 3, 39 of the 50 miRNA candidate genes were included in dbDEMC database and 21 candidates were included in miRCancer database. This suggests that these miRNAs are abnormally expressed in breast cancer and are associated with breast cancer.

PhenomiR database [45] contains miRNAs differentially expressed in diseased tissues compared to normal tissues. Twenty-six candidate miRNAs are present in PhenomiR database, indicating that they are upregulated or downregulated in breast cancer. Although hsa-mir-4480 [41] had a centrality score of 9. It was still described as a breast cancer-related miRNA in the SKBR3 network. Hsa-mir-885 [40] directly targets B7-H3 by association with the B7-H3 3′-UTR region, suggesting that hsa-mir-885 have a direct role in modulating B7-H3 protein expression in breast cancer. Chaluvally-Raghavan et al. [42] demonstrated that hsa-miR-569, which is overexpressed in a subset of ovarian and breast cancers, at least in part owing to the 3q26.2 amplicon, alters cell survival and proliferation. Xian Wang et al. [43] performed a differential expression profile analysis of hsa-mir-4454 in breast cancer cells. Junjun et al. [44] confirmed that hsa-mir-3135b is differentially expressed in the breast cancer cell line MCF7. Hsa-mir-6838 is marked “Unconfirmed” and thus not currently supported by the databases and the relevant literature.

Supplementary Table S1 lists the top 50 candidates associated with lung cancer. DbDEMC database contains 35 candidates showing abnormal expression in lung cancer, and 31 candidate miRNAs are present in miRCancer database, demonstrating their association with lung cancer disease. Thirty-seven candidate miRNAs are present in PhenomiR database, showing their expression levels significantly altered in lung cancer cells. NCIH460, a lung cancer cell line, was treated with a screening library, revealing the ability of hsa-mir-4480 [46] to inhibit the growth of lung cancer cells. Park et al. [47] showed that hsa-mir-1843 is significantly upregulated compared with normal lung tissue. Long noncoding RNA NEAT1 promotes non-small cell lung cancer progression through regulation of the hsa-mri-4262 pathway [48]. In addition, EZH2 and miR-4448 show mutual negative regulations for tumor progression via epithelial mesenchymal transition in small cell lung cancer [49]. Hsa-mir-3161 is listed as differentially expressed miRNAs in lung adenocarcinoma by Gou et al. [50]. Hsa-mir-3074-5p is also significantly correlated with small cell lung cancer metastasis [51].

For pancreatic cancer, the top 50 candidate associations are listed in Supplementary Table S2. Forty-eight and 18 candidates are present in dbDEMC and miRCancer databases, respectively, indicating that they are associated with the disease. Forty candidate miRNAs are present in the PhenomiR database, suggesting that the expression levels of this gene in pancreatic cancer cells significantly differ from those in normal tissues.

The data of disease and miRNA used herein was derived from the latest Human miRNA–Disease Database (HMDD, released in March 2019) [52], which contains 7908 miRNA–disease association pairs that have been validated by biological experiments. Disease terms from the American Medical Library (Mesh, hattp://www.ncbi.nlm.nih.gov/mesh) were used to construct directed acyclic graphs (DAGs) to calculate the semantic similarities of the disease. We obtained the disease phenotypic similarity [53] information from previous work. The information of 530 miRNA families is extracted from miRBase (version 22.1) [54]. According to previous studies, we obtained 1309 clusters by setting the distance between two miRNAs to no more than 20 kb.

**Figure 3:**
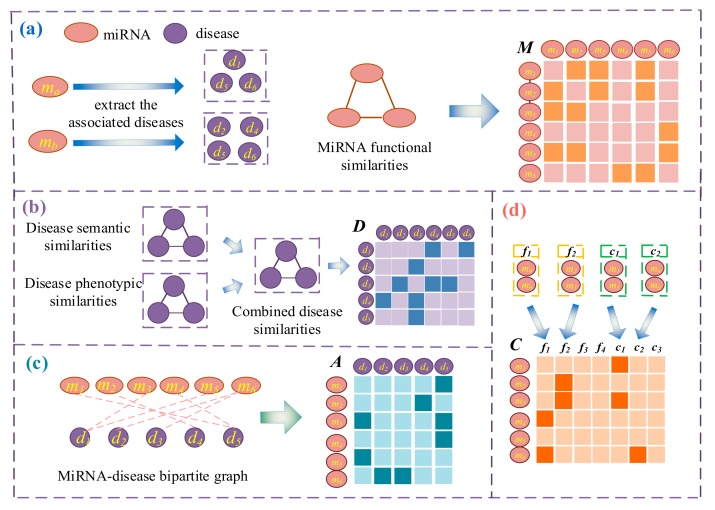
Multiple data representations of miRNAs and diseases: (**a**) calculate miRNA similarities through miRNA–associated diseases, (**b**) calculate the similarities of disease by combining disease semantic similarities and disease phenotypic similarities, (**c**) establish association matrix A based on known associations between miRNAs and diseases, and (**d**) create a representation matrix of miRNA families and clusters.

The primary goal of the study was to predict disease–miRNA associations. To integrate miRNA similarities, disease similarities, miRNA–disease association, and miRNA node attributions, a model based on nonnegative matrix factorization was constructed (Figure 3), and then this model was solved with an iterative algorithm. This model can reveal association scores of miRNAs $m_{i}$ and diseases $d_{j}$. A higher association score indicates a greater likelihood of an association.

## 3. Materials and Methods

### 3.1. Data Representation of miRNAs and Diseases

***MiRNA similarities.*** It is well-known that miRNAs with similar functions are often associated with similar diseases. Wang et al. [19] successfully calculated miRNA similarities by using miRNA-associated diseases. For instance, diseases $d_{1},\;d_{5},\;d_{6}$ are associated with miRNA $m_{a}$, while diseases $d_{2},d_{4},d_{5},d_{6}$ are associated with miRNA $m_{b}$ and the similarity $M\left(m_{a},m_{b}\right)$ of $S_{a}=\left\{d_{1},d_{5},d_{6}\right\}$ and $S_{b}=\left\{d_{2},d_{4},d_{5},d_{6}\right\}$ is calculated as the similarity of $m_{a}$ and $m_{b}$ (Figure 3a). The miRNA similarity matrix is $M=\left[M_{ij}\right]\in {ℜ}^{N_{m}\times N_{m}}$, where $N_{m}$ is the number of miRNAs and $M_{ij}$ is the similarity of $m_{i}$ and $m_{j}$. Generally, $M_{ij}$ is more than or equal to 0; a higher score indicates greater similar between $m_{i}$ and $m_{j}$.

***Disease similarities***. From the dual perspectives of disease semantics and phenotypes (signs and symptoms), we measured the similarity of two diseases. Generally, we used a DAG to represent disease-related semantic terms. A larger number of common terms on the DAG for two diseases reflects greater similarity between the two diseases. If the two diseases have more common phenotypes, then the two diseases are more similar. Therefore, we quantified the similarities of diseases based on the semantics and phenotype of the disease (Figure 3b). Xuan et al. [21,23,31,55] successfully integrated this information and calculated the similarity of diseases, which we obtained from the previous method. The similarity matrix $D=\left[D_{ij}\right]\in {ℜ}^{N_{d}\times N_{d}}$ containing $N_{d}$ diseases indicates the similarity of disease $d_{i}$ and disease $d_{j}$; a larger value indicates greater similar, and the value of $D_{ij}$ is generally between 0 and 1.

***MiRNA–disease associations.*** According to the known associations between miRNAs and diseases, an associations matrix $A=\left[A_{ij}\right]\in {ℜ}^{N_{m}\times N_{d}}$ was constructed (Figure 3c). Each row of the association matrix A corresponds to a miRNA, of which the column corresponds to a disease. If the miRNA $m_{i}$ is associated with a disease $d_{j}$, then $A_{ij}=1$. If $m_{i}$ and $d_{j}$ are not associated or no association has been observed so far, then $A_{ij}=0$.

***MiRNA node attributes.***$C\in {ℜ}^{N_{m}\times \left(N_{f}+N_{c}\right)}$ is a miRNA family and cluster characteristic matrix, with the rows representing miRNAs and columns showing family or cluster information (Figure 3d). Vector $C_{i}$ represent miRNA $m_{i}$ subordinate to $N_{f}$ family and $N_{c}$ cluster, which are considered node attributes. $C_{ij}\left(C_{i\left(N_{f}+k\right)}\right)=1$ indicates that the miRNA belongs to the $j^{th}$ family or $k^{th}$ cluster; otherwise, the value is 0.

### 3.2. Prediction Models for Disease–miRNA Associations

A model based on nonnegative matrix factorization was constructed, which integrates miRNA similarities, disease similarities, miRNA and disease associations, as well as miRNA family and cluster information. Let $U\in {ℜ}^{N_{m}\times N_{d}}$ indicate the predicted miRNA associated score with the disease. $N_{m}$ is the number of miRNAs, $N_{d}$ is the number of diseases, and $U_{ij}$ is the score of the miRNA and disease association. A larger score means that $m_{i}$ and $d_{j}$ are more likely to be associated, and $U_{ij}$ is typically greater than or equal to 0.

***Projection of miRNA,disease, and node attributes.*** We projected miRNA disease-related information into low-dimensional space to extract representative low-dimensional feature vectors. For the miRNA, M denotes the miRNA similarities matrix, which is projected into the c-dimensional space. $X\in {ℜ}^{N_{m}\times c}$ is a projection matrix of miRNA similarities, $MX\in {ℜ}^{N_{m}\times c}$ represents the low-dimensional feature matrix of the miRNAs, and the $i^{th}$ row of MX represents the low-dimensional feature vector about $m_{i}$.

For the disease, D is the similarities matrix of the disease, which can be projected into the low-dimensional space, and the low-dimensional feature matrix can be obtained. $Y\in {ℜ}^{N_{d}\times c}$ is a projection matrix of disease similarities, $DY\in {ℜ}^{N_{d}\times c}$ is the low-dimensional feature matrix of the disease, and the $j^{th}$ row of DY represents the low-dimensional feature vector about $d_{j}$.

For the miRNA of the node attributes, $C\in {ℜ}^{N_{m}\times \left(N_{f}+N_{d}\right)}$ is the feature matrix of the family and cluster, which is projected into the low-dimensional space to obtain the low-dimensional feature matrix of the node attributes of the miRNA. $Z\in {ℜ}^{\left(N_{f}+N_{c}\right)\times c}$ is the projection matrix of the node attributes. $CZ\in {ℜ}^{N_{m}\times c}$ is a miRNA low-dimensional feature matrix with node attributes, and its $i^{th}$ row is a low-dimensional feature vector of the miRNA family and cluster.

***Modelling miRNA-disease associations.*** In association matrix A, the values of all 1 represent the observed miRNA disease association, 0 indicates that an association has not been observed, and most values of 0 indicate that the miRNA is not associated with the disease. The association matrix A reflects the true associations between miRNAs and diseases. The element $U_{ij}$ in the score matrix U indicates the possibility that the miRNA is associated with a disease. The evaluated score matrix U should be as consistent as possible with the actual correlation. The objective function is obtained as follows,

(3) $$\underset{U\geq 0}{min}{\left\|U-A\right\|}_{F}^{2},$$

where ${\left\|\cdot \right\|}_{F}$ is the Frobenius norm of a matrix.

***Modelling similarities of miRNAs and diseases.*** The $i^{th}$ row of the low-dimensional feature matrix $\left(MX\right)\in {ℜ}^{N_{m}\times c}$ represents the feature vectors of the miRNA $m_{i}$ in the c-dimensional space. Similarly, the $j^{th}$ column of ${\left(DY\right)}^{T}\in {ℜ}^{c\times N_{d}}$ represents the feature vector of the disease $d_{j}$ in the c-dimensional space. The closer the miRNA $m_{i}$ is to the disease $d_{j}$ in the c-dimensional space, i.e., the larger the value of ${\left(MX\right)}_{i}{\left(DY\right)}_{j}^{T}$, the more likely $m_{i}$ is associated with $d_{j}$. An element of the score matrix $U_{ij}$ denotes the probability that the predicted $m_{i}$ is associated with $d_{j}$. $U_{ij}$ and ${\left(MX\right)}_{i}{\left(DY\right)}_{j}^{T}$ should be as consistent as possible. An objective function expansion was obtained as follows,

(4) $$\underset{U\geq 0}{min}\|U-A{\|}_{F\;}^{2}+\alpha_{1}\|U-MX{\left(DY\right)}^{T}{\|}_{F\;}^{2},$$

where $\alpha_{1}$ is a hyperparameter for adjusting the contribution of the second section.

***Modelling node attributes of miRNAs.***${\left(CZ\right)}_{i}$ is the $i^{th}$ row of the matrix $\left(CZ\right)\in {ℜ}^{N_{m}\times c}$, which records the low-dimensional feature vector of $m_{i}$ based on the miRNA and node attribution. Correspondingly, ${\left(DY\right)}_{j}^{T}$ is the $ij^{th}$ row of the matrix of ${\left(DY\right)}^{T}$, which records the low-dimensional feature vector. The more consistent ${\left(CZ\right)}_{i}$ and ${\left(DY\right)}_{j\;}^{T}$, the more likely $m_{i}$ is associated with $d_{j}$. $U_{ij}$ is the estimated association score of $m_{i}$ and $d_{j}$. To make the predicted score matrix U and actual calculated association as consistent as possible, our objective function is expanded,

(5) $$\underset{U\geq 0}{min}\|U-A{\|}_{F}^{2}+\alpha_{1}\|U-MX{\left(DY\right)}^{T}{\|}_{F}^{2}+\alpha_{2}\|U-CZ{\left(DY\right)}^{T}{\|}_{F}^{2},$$

where $\alpha_{2}$ is the contribution of the adjustment node attribute information.

***Modelling the topological structure of miRNAs.*** miRNAs and *k* neighbours are more likely to be associated with similar diseases. A graph model S based on similarity between miRNA and miRNA was created,

(6) $$S_{ij}=\left\{\begin{array}{c} 1,\;\mathrm{if}\;\mathrm{miRNA}\;m_{i\;}\mathrm{is}\;\mathrm{one}\;\mathrm{of}\;\mathrm{the}\;\mathrm{similar}\;\mathrm{neighbours}\;\mathrm{of}\;\mathrm{miRNA}\;m_{j}\\ 0,\;\mathrm{otherwise} \end{array}\right..$$

The graph Laplacian matrix L of miRNA feature graph S is defined as follows,

(7) L=W−S,

where W is a diagonal matrix with $W\left(i,i\right)=\overset{N_{m}}{\underset{j}{\sum }}S\left(i,j\right)$. Graph models are used to introduce smooth regularization, as miRNA with similar features should have similar diseases. The graph model is used to reflect the correlation and similarity of known indications between different miRNAs. The objective function is expanded as follows,

(8) $$\underset{U\geq 0}{min}\|U-A{\|}_{F}^{2}+\alpha_{1}\|U-MX{\left(DY\right)}^{T}{\|}_{F}^{2}+\alpha_{2}\|U-CZ{\left(DY\right)}^{T}{\|}_{F}^{2}+\alpha_{3}Tr\left(U^{T}LU\right),$$

where $\alpha_{3}$ is a hyperparameter that adjusts the contribution of the regularization of graphs to the entire objective function and Tr() represents the trac of the matrix.

Consider the sparseness of associations. Since a disease is only associated with a limited number of miRNAs, we imposed $l_{1}$-regularization to U learn sparse associations. The objective function is expanded as follows,

(9) $$\underset{U\geq 0}{min}\|U-A{\|}_{F}^{2}+\alpha_{1}\|U-MX{\left(DY\right)}^{T}{\|}_{F}^{2}+\alpha_{2}\|U-CZ{\left(DY\right)}^{T}{\|}_{F}^{2}+\alpha_{3}Tr\left(U^{T}LU\right)+\alpha_{4}\|U{\|}_{1}.$$

### 3.3. Optimization

The objective function $L\left(U,X,Y,Z\right)$ in Equation (9) is a non-convex function, and it is impractical to obtain its global optimal solution. We divided the function into four subproblems to obtain a near-optimal solution for $L\left(U\right)$.

U**-subproblem**. When X,Y, and Z are fixed, the subproblem for solving U is as follows,

(10) $$\begin{array}{c} \underset{U\geq 0}{\min}L\left(U\right)=\|U-A{\|}_{F}^{2}+\alpha_{1}\|U-MX{\left(DY\right)}^{T}{\|}_{F}^{2}\\ +\alpha_{2}\|U-CZ{\left(DY\right)}^{T}{\|}_{F}^{2}+\alpha_{3}Tr\left(U^{T}LU\right)+\alpha_{4}\|U{\|}_{1}. \end{array}$$

According to the trace property and Frobenius norm of the matrix, $L\left(U\right)$ can be rewritten as follows,

(11) $$\begin{array}{ll} \underset{U\geq 0}{min}L\left(U\right) & =\|U-A{\|}_{F}^{2}+\alpha_{1}\|U-MX{\left(DY\right)}^{T}{\|}_{F}^{2}+\alpha_{2}\|U-CZ{\left(DY\right)}^{T}\|+\alpha_{3}Tr\left(U^{T}LU\right)+\alpha_{4}\|U{\|}_{1}\\ & =Tr\left(UU^{T}-UA^{T}-AU^{T}+AA^{T}\right)\\ & \;\;+\alpha_{1}Tr(UU-UDY{\left(MX\right)}^{T}-MX{\left(DY\right)}^{T}U^{T}+MX{\left(DY\right)}^{T}DY\left(MX{)}^{T}\right)\\ & \;\;\;+\alpha_{2}Tr(UU^{T}-UDY{\left(CZ\right)}^{T}-CZ{\left(DY\right)}^{T}U^{T}+CZ{\left(DY\right)}^{T}DY\left(CZ{)}^{T}\right)\\ & \;\;+\alpha_{3}Tr\left(U^{T}WU-U^{T}SU\right)+\alpha_{4}{\left\|U\right\|}_{1}, \end{array}$$

where Tr() is the trace of the matrix. By setting the derivative of $L\left(U\right)$ with respect to U to 0, we obtain the following equation,

(12) $$2U-2A+2\alpha_{1}U-2\alpha_{1}MX{\left(DY\right)}^{T}+2\alpha_{2}U-2\alpha_{2}CZ{\left(DY\right)}^{T}+2\alpha_{3}WU-2\alpha_{3}SU+\alpha_{4}B=0,$$

where $B=\left[B_{ij}\right]\in ℜ{\;}^{N_{m}\times N_{d}}$ is a matrix of which the elements are all 1. By multiplying both sides of Equation (12) by $U_{ij}$, we obtain the following equation

(13) $$\begin{array}{c} (2U-2A+2\alpha_{1}U-2\alpha_{1}MX{\left(DY\right)}^{T}+2\alpha_{2}U\\ -2\alpha_{2}CZ{\left(DY\right)}^{T}+2\alpha_{3}WU-2\alpha_{3}SU+\alpha_{4}B{)}_{ij}U_{ij}=0. \end{array}$$

Finally, according to the coordinate descent algorithm, we can obtain $U_{ij}$’s updated formula by multiplying its current value with the ratio of the negative terms to the positive term of Equation (13),

(14) $$U_{ij}^{new}\leftarrow U_{ij}\cdot \frac{2A+2\alpha_{1}MX{\left(DY\right)}^{T}+2\alpha_{2}CZ{\left(DY\right)}^{T}+2\alpha_{3}SU}{2U+2\alpha_{1}U+2\alpha_{2}U+2\alpha_{3}WU+\alpha_{4}B}.$$

**X-subproblem**. When U,Y, and Z are fixed, the subproblem for solving X is,

(15) $$\underset{X\geq 0}{min}L\left(X\right)=\alpha_{1}\|U-MX{\left(DY\right)}^{T}{\|}_{F}^{2}.$$

According to the trace property and Frobenius norm of the matrix, $L\left(X\right)$ can be rewritten as,

(16) $$L\left(X\right)=\alpha_{1}Tr(UU^{T}-UDY{\left(MX\right)}^{T}-MX{\left(DY\right)}^{T}U^{T}+MX{\left(DY\right)}^{T}DY\left(MX{)}^{T}\right).$$

By setting the derivative of $L\left(X\right)$ with respect to X to 0, we obtain the following equation,

(17) $$-2\alpha_{1}M^{T}UDY+2\alpha_{1}M^{T}MX{\left(DY\right)}^{T}DY=0.$$

By multiplying both sides of Equation (17) by $X_{ij}$, we obtain the following equation,

(18) $${\left(-2\alpha_{1}M^{T}UDY+2\alpha_{1}M^{T}MX{\left(DY\right)}^{T}DY\right)}_{ij}X_{ij}=0.$$

X’s updating rule by applying the coordinate gradient descent algorithm is as follows,

(19) $$X_{ij}^{new}\leftarrow X_{ij}\cdot \frac{M^{T}UDY}{M^{T}MX{\left(DY\right)}^{T}DY}.$$

Y**-subproblem**. When U,X, and Z are fixed, the subproblem for solving Y is as follows,

(20) $$L\left(Y\right)=\alpha_{1}\|U-MX{\left(DY\right)}^{T}{\|}_{F}^{2}+\alpha_{2}\|U-CZ{\left(DY\right)}^{T}\|.$$

We transformed the Frobenius norms of the matrices in $L\left(Y\right)$ to their trace norms and rewrote $L\left(Y\right)$ as follows,

(21) $$\begin{array}{ll} L\left(Y\right) & =\alpha_{1}\|U-MX{\left(DY\right)}^{T}{\|}_{F}^{2}+\alpha_{2}\|U-CZ{\left(DY\right)}^{T}\|\\ & =\alpha_{1}Tr(UU^{T}-UDY{\left(MX\right)}^{T}-MX{\left(DY\right)}^{T}U^{T}+MX\left(DY{)}^{T}DYX^{T}M^{T}\right)\\ & \;+\alpha_{2}Tr(UU^{T}-UDY{\left(CZ\right)}^{T}-CZ{\left(DY\right)}^{T}U^{T}+CZ\left(DY{)}^{T}DYZ^{T}C\right). \end{array}$$

By setting the derivative of $L\left(Y\right)$ with respect to 0, we obtain the following,

(22) $$2\alpha_{1}D^{T}DY{\left(MX\right)}^{T}MX-2\alpha_{1}D^{T}U^{T}MX+2\alpha_{2}D^{T}DY{\left(CZ\right)}^{T}CZ-2\alpha_{2}D^{T}U^{T}CZ=0.$$

After both sides of Equation (22) are multiplied by ${\left(Y\right)}_{ij}$, we obtain the following equation,

(23) $$(2\alpha_{1}D^{T}DY{\left(MX\right)}^{T}MX-2\alpha_{1}D^{T}U^{T}MX+2\alpha_{2}D^{T}DY{\left(CZ\right)}^{T}CZ-2\alpha_{2}D^{T}U^{T}CZ{)}_{ij}Y_{ij}=0.$$

Y’s updating rule by applying the coordinate gradient descent algorithm is as follows,

(24) $$Y_{ij}^{new}\leftarrow Y_{ij}\cdot \frac{\alpha_{1}D^{T}U^{T}MX+\alpha_{2}D^{T}U^{T}CZ}{\alpha_{1}D^{T}DY{\left(MX\right)}^{T}MX+\alpha_{2}D^{T}DY{\left(CZ\right)}^{T}CZ}.$$

Z**-subproblem.** When U,X, and Y are fixed, the subproblem for solving Z is as follows,

(25) $$\underset{Z\geq 0}{min}L\left(Z\right)=\alpha_{2}\|U-CZ{\left(DY\right)}^{T}{\|}_{F}^{2}.$$

Similar to the process for solving the subproblems of U,X, and Y, $L\left(Z\right)$ is transformed first according to the characteristic of the matrix traces. The derivative is then determined with respect to Z. Finally, the gradient descent algorithm is applied to obtained the updated rule for Z,

(26) $$Z_{ij}^{new}\leftarrow Z_{ij}\cdot \frac{C^{T}UDY}{C^{T}CZ{\left(DY\right)}^{T}DY}.$$

**Figure 4:**
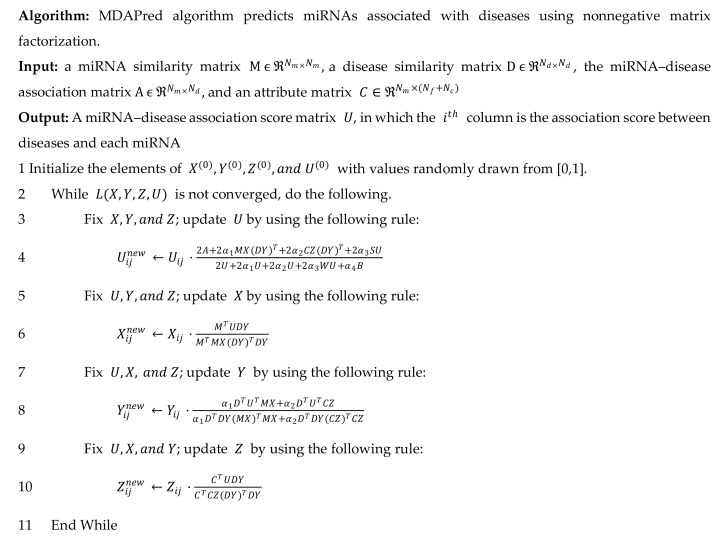
Iterative algorithm for estimation of the miRNA–disease association scores.

The iterative process is over when the absolute difference of $L\left(U,X,Y,Z\right)$ at two adjacent moments is less than a threshold (ε = 10^−6^) or when the maximum number of iterations, 100, is reached. Finally, $U_{ij}$ is regarded as the estimated association score between miRNA $m_{i}$ and disease $d_{j}$ (Figure 4). 

## 4. Conclusions

In the current study, MDAPred, a new method based on nonnegative matrix factorization, was developed for predicting potential disease–miRNA candidates. MDAPred deeply integrates the projections of multiple kinds of connecting edges and the node attributions of miRNAs to enhance the detection of the disease–miRNA associations. MDAPred also takes full advantage of information about the neighbours of miRNAs to capture the local topology of miRNAs. A sparse penalty was introduced to improve the performance of MDAPred. An iterative algorithm was proposed to obtain discriminative ability. MDAPred was superior to other tested methods not only in their AUCs but also in their AUPRs. Additionally, MDAPred is useful for biologists, as it can list more real disease–miRNA associations in its top ranking list. Case studies of three diseases revealed the ability of MDAPred to identify potential candidates. Therefore, MDAPred can serve as a prioritization tool for identifying real associations of disease miRNAs through wet-lab experiments.

## Figures and Tables

**Table 1:** Areas under the ROC curves (AUCs) of MDAPred and other methods on 15 diseases.

Disease Name	AUC					
	MDAPred	DMPred	PBMDA	GSTRW	Liu’s Method	BNPMDA
Breast neoplasms	**0.986**	0.974	0.906	0.837	0.920	0.902
Hepatocellular carcinoma	**0.982**	0.931	0.910	0.791	0.929	0.900
Glioma	**0.957**	0.855	0.882	0.786	0.914	0.843
Acute myeloid leukemia	**0.979**	0.963	0.885	0.796	0.910	0.865
Lung neoplasms	**0.964**	0.944	0.862	0.813	0.906	0.855
Melanoma	**0.978**	0.910	0.849	0.758	0.893	0.839
Osteosarcoma	0.968	**0.985**	0.860	0.771	0.897	0.859
Ovarian neoplasms	**0.970**	0.967	0.888	0.844	0.918	0.877
Pancreatic neoplasms	**0.956**	0.821	0.879	0.833	0.902	0.870
Alzheimer Disease	**0.968**	0.958	0.833	0.816	0.875	0.830
Carcinoma, Renal Cell	**0.964**	0.894	0.856	0.784	0.900	0.854
Diabetes Mellitus, Type 2	**0.964**	0.936	0.870	0.870	0.905	0.869
Glioblastoma	0.938	**0.951**	0.849	0.759	0.889	0.843
Heart failure	**0.962**	0.959	0.884	0.814	0.909	0.882
Atherosclerosis	**0.962**	0.955	0.891	0.822	0.910	0.876
Average AUC	**0.964**	0.933	0.873	0.806	0.904	0.839

The bold values indicate the higher AUCs.

**Table 2:** AUPRs of MDAPred and other methods on 15 diseases.

Disease Name	AUPR					
	MDAPred	DMPred	PBMDA	GSTRW	Liu’s Method	BNPMDA
Breast neoplasms	**0.818**	0.800	0.718	0.389	0.725	0.566
Hepatocellular carcinoma	**0.816**	0.715	0.767	0.483	0.749	0.676
Glioma	**0.613**	0.175	0.390	0.224	0.436	0.386
Acute myeloid leukemia	**0.544**	0.466	0.386	0.122	0.408	0.324
Lung neoplasms	**0.686**	0.620	0.561	0.370	0.596	0.542
Melanoma	**0.689**	0.366	0.482	0.205	0.524	0.491
Osteosarcoma	0.601	**0.620**	0.356	0.181	0.373	0.327
Ovarian neoplasms	**0.714**	0.366	0.529	0. 400	0.236	0.496
Pancreatic neoplasms	**0.692**	0.569	0.457	0.333	0.556	0.478
Alzheimer Disease	**0.522**	0.351	0.136	0.086	0.485	0.220
Carcinoma, Renal Cell	**0.481**	0.206	0.314	0.135	0.143	0.299
Diabetes Mellitus, Type 2	**0.549**	0.398	0.259	0.132	0.356	0.268
Glioblastoma	**0.533**	0.284	0.346	0.161	0.303	0.336
Heart failure	**0.599**	0.393	0.301	0.134	0.348	0.300
Atherosclerosis	**0.315**	0.309	0.304	0.084	0.297	0.218
Average PR	**0.603**	0.500	0.436	0.233	0.463	0.359

The bold values indicate the higher AUPRs.

**Table 3:** Comparison of different methods based on AUCs with a paired *t*-test.

*p-*Value between MDAPred and Other Methods	DMPred	PBMDA	GSTRW	Liu’s Method	BNPMDA
*p*-values of ROC curves	2.4983 × 10^−^^41^	3.2311 × 10^−^^5^	6.3212 × 10^−^^16^	6.9812 × 10^−^^8^	2.9742 × 10^−^^6^
*p*-values of PR curves	2.2341 × 10^−^^35^	1.8643 × 10^−^^6^	1.6542 × 10^−^^6^	3.4521 × 10^−^^5^	8.8432 × 10^−^^4^

**Table 4:** The top 50 breast cancer-related candidates.

Rank	MiRNA name	Evidence	Rank	MiRNA name	Description
1	hsa-mir-186	dbDEMC, PhenomiR	26	hsa-mir-885	literature [40]
2	hsa-mir-99b	dbDEMC, PhenomiR	27	hsa-mir-6838	Unconfirmed
3	hsa-mir-483	PhenomiR	28	hsa-mir-323a	dbDEMC, PhenomiR
4	hsa-mir-4480	literature [41]	29	hsa-mir-1244	dbDEMC
5	hsa-mir-181d	dbDEMC, PhenomiR, miRCancer	30	hsa-mir-361	PhenomiR, miRCancer
6	hsa-mir-28	dbDEMC, PhenomiR	31	hsa-mir-216a	dbDEMC, PhenomiR, miRCancer
7	hsa-mir-455	PhenomiR, miRCancer	32	hsa-mir-136	dbDEMC, PhenomiR
8	hsa-mir-154	dbDEMC, PhenomiR, miRCancer	33	hsa-mir-569	literature [42]
9	hsa-mir-330	dbDEMC, PhenomiR, miRCancer	34	hsa-mir-336	dbDEMC
10	hsa-mir-454	dbDEMC, PhenomiR	35	hsa-mir-325	dbDEMC, PhenomiR
11	hsa-mir-181	dbDEMC, PhenomiR, miRCancer	36	hsa-mir-571	dbDEMC
12	hsa-mir-208b	dbDEMC, PhenomiR	37	hsa-mir-95	dbDEMC, PhenomiR
13	hsa-mir-663	dbDEMC, PhenomiR	38	hsa-mir-517b	dbDEMC, PhenomiR, miRCancer
14	hsa-mir-133	dbDEMC, PhenomiR, miRCancer	39	hsa-mir-323	dbDEMC, PhenpmiR
15	hsa-mir-30	dbDEMC, PhenomiR, miRCancer	40	hsa-mir-633	dbDEMC
16	hsa-mir-504	dbDEMC	41	hsa-mir-1183	dbDEMC
17	hsa-mir-543	dbDEMC	42	hsa-mir-4454	literature [43]
18	hsa-mir-217	dbDEMC, PhenomiR, miRCancer	43	hsa-mir-705	dbDEMC
19	hsa-mir-33	dbDEMC, PhenomiR, miRCancer	44	hsa-mir-532	dbDEMC, PhenomiR
20	hsa-mir-211	dbDEMC, PhenomiR, miRCancer	45	hsa-mir-126a	dbDEMC, miRCancer
21	hsa-mir-449b	dbDEMC, PhenomiR, miRCancer	46	hsa-mir-1909	dbDEMC
22	hsa-mir-362	miRCancer	47	hsa-mir-539	dbDEMC, PhenomiR, miRCancer
23	hsa-mir-208	dbDEMC, PhenomiR	48	hsa-mir-520f	PhenomiR, miRCancer
24	hsa-mir-433	dbDEMC, PhenomiR, miRCancer	49	hsa-mir-498	miRCancer
25	hsa-mir-520e	dbDEMC, PhenomiR, miRCancer	50	hsa-mir-3135b	literature [44]

## Supplementary Materials

The following are available online. Table S1: The top 50 candidates for lung cancer. Table S2: The top 50 candidates for pancreatic cancer. Table S3: The top 50 potential candidates for 341 diseases.
